# 2-(4-Methylthiazol-5-yl) Ethyl Nitrate Hydrochloride Ameliorates Cognitive Impairment via Modulation of Oxidative Stress and Nuclear Factor Kappa B (NF-κB) Signaling Pathway in Chronic Cerebral Hypoperfusion-Associated Spontaneously Hypertensive Rats

**DOI:** 10.3390/antiox13050585

**Published:** 2024-05-10

**Authors:** Jiang Li, Shaofeng Xu, Ling Wang, Xiaoliang Wang

**Affiliations:** State Key Laboratory of Bioactive Substances and Functions of Natural Medicines, Institute of Materia Medica, Chinese Academy of Medical Sciences & Peking Union Medical College, Beijing 100050, China; lijiang@imm.ac.cn (J.L.); xusf@imm.ac.cn (S.X.); wangl@imm.ac.cn (L.W.)

**Keywords:** 2-(4-methylthiazol-5-yl) ethyl nitrate hydrochloride (W1302), nitric oxide donor, hypertension, vascular dementia, oxidative stress, NF-κB pathway

## Abstract

Hypertension reduces the bioavailability of vascular nitric oxide (NO) and contributes to the onset of vascular dementia (VaD). A loss of NO bioavailability increases inflammation and oxidative stress. 2-(4-Methylthiazol-5-yl) ethyl nitrate hydrochloride (W1302) is a novel nitric oxide donor (NOD) which is undergoing phase I clinical trials in China for the treatment of VaD. In this study, we investigated the protective effects of W1302 in VaD rats induced by the permanent occlusion of a bilateral common carotid arteries model related to spontaneous hypertension (SHR-2VO), and we further explored the underlying mechanisms. Nimodipine was used as a positive control. Our results showed that W1302 treatment for 4 weeks (10 mg/Kg/day) exhibited stronger improvement in the spatial learning and memory deficits in SHR-2VO rats compared with nimodipine with slightly lower systolic blood pressure (SBP). Meanwhile, W1302 treatment significantly increased NO and cGMP production, restored mitochondrial membrane potential and attenuated oxidative stress as evidenced by increasing ATP production and reducing malondialdehyde (MDA) levels in the brain. Furthermore, W1302 treatment markedly inhibited the iNOS activity and decreased TNF-α expression via inhibiting the nuclear factor kappa B (NF-κB) signaling pathway. Nimodipine treatment also restored these aberrant changes, but its ATP production was weaker than that of W1302, and there was no significant effect on NO release. Taken together, W1302 exhibited beneficial effects on complications in VaD with hypertension, which is involved in suppressing oxidative damage, and the inflammatory reaction might be mediated by an increase in NO release. Therefore, W1302 has therapeutic potential for the treatment of VaD caused by chronic cerebral hypoperfusion-associated spontaneous hypertension.

## 1. Introduction

Vascular dementia (VaD) is the second most common form of dementia caused by vascular dysfunction [1]. There is an accumulating body of evidence suggesting that hypertension increases the risk of VaD [1,2]. According to the World Health Organization, the hypertension prevalence ranged from 13.0% to 41.0% in 2015 for 182 countries, and about 30% of the population over the age of 60 years had the disease [3]. Also, patients with hypertension (SBP ≥ 160 mmHg) were associated with a 4.8-fold higher risk of dementia onset compared to the normotensive individuals [4,5]. Hypertension has been shown to reduce the bioavailability of vascular nitric oxide (NO) as well as produce inflammation and oxidative stress [6,7,8,9,10]. The loss of NO bioavailability plays a key role in the development and progression of VaD [1,2]. Therefore, providing an exogenous source of NO is regarded as a promising strategy for the treatment of VaD.

2-(4-Methylthiazol-5-yl) ethyl nitrate hydrochloride (W1302) is a novel nitric oxide donor (NOD) derived from clomethiazole (CMZ), and it is under development as a therapeutic drug for Alzheimer’s disease (AD) [11,12,13,14,15]. And the clinical trials of W1302 for the treatment of VaD were also approved by the Chinese National Medical Products Administration (NMPA) in 2023. W1302 has been verified as effective against Aβ, glutamate or oxygen-glucose deprivation (OGD)-induced neuronal damage in vitro [11,12,13,14,15]. In vivo, W1302 also showed neuroprotective effects in the treatment of Alzheimer’s disease (AD) by activating NO- and GABA-dependent signaling in MK-801 [12] or scopolamine [13] impaired mice, stimulating an NO/sGC/cGMP signal transduction cascade in rats after forebrain cholinergic depletion [14], reducing neurotoxic forms of Aβ or tau protein, restoring neuronal plasticity, antioxidant activity, and anti-neuroinflammation, and improving long-term potentiation (LTP) via CREB/BNDF signaling in a novel sporadic model of Alzheimer’s disease and multiple familial mouse models [15]. In addition, the neuroprotective effect of W1302 was also partially dependent on the potentiation of GABA_A_ receptors [16]. These results suggested that W1302 might have a therapeutic effect on VaD. However, there is no study to date on the effects of W1302 on VaD outcomes in chronic cerebral hypoperfusion-associated spontaneous hypertension.

The permanent occlusion of bilateral common carotid arteries in spontaneously hypertensive (SHR-2VO) rats is an ideal animal model that mimics clinical chronic cerebral hypoperfusion related to hypertension following lacunar stroke and cerebral small vessel disease, including endothelial dysfunction, a reduction in NO bioavailability, neuroinflammation, oxidative stress, and subsequent cognitive impairment [17,18]. Therefore, in this study, we investigated whether the long-term administration of W1302 could improve the cognitive impairment in SHR-2VO rats. Meanwhile, the possible neuroprotective mechanisms of W1302, including the restoration of mitochondrial function and the inhibition of oxidative stress, inflammation and the NF-κB pathway were investigated. Previous studies showed that nimodipine, an L-type calcium channel antagonist, exerts neuroprotective effects via regulating the NF-κB signaling pathway in rats after focal cerebral ischemia injury, so it served as a positive control in this study [19].

## 2. Materials and Methods

### 2.1. Animals

Five-month-old male spontaneously hypertensive rats (SHRs, *n* = 81) and age-matched Wistar Kyoto (WKY) rats (*n* = 16) were purchased from Vital River Laboratories Technology Co., Ltd. (Beijing, China). During the experimental period, the animals were housed in a room maintained at 23 ± 2 °C with a 12 h light/dark cycle and fed a standard rodent diet with water ad libitum. All animal experiments were approved by the Animal Care and Use Committee of the Peking Union Medical College and the Chinese Academy of Medical Sciences (Beijing, China; approval number: 00005007).

### 2.2. Surgery

The model of chronic cerebral hypoperfusion in spontaneously hypertensive rats (SHR-2VO rats) was established by permanent bilateral carotid artery occlusion surgery, as described previously [20]. The WKY rats underwent the same procedure without vascular ligation.

### 2.3. Drug Administration and Experimental Design

On day 3 after surgery, the survived SHR-2VO rats (*n* = 45) were randomly divided into three groups: the SHR-2VO group (*n* = 15), the W1302 group (*n* = 15), and the nimodipine group (*n* = 15). Treated groups received W1302 (10 mg/Kg/day, synthesized by Institute of Materia Medica, Beijing, China with a purity of higher than 99%) or nimodipine (10 mg/Kg/day, Hunan Baicao Pharmaceutical Co., Ltd., Yongzhou, China) by oral gavage. WKY rats (*n* = 16) were employed as non-hypertensive sham-operation control by oral gavage distilled water. All treatment continued for 4 weeks beginning at 5 months old. After 3 weeks of administration, spatial learning and memory abilities of rats were evaluated with a Morris water maze. Subsequently, the systolic blood pressure and heart rate of conscious rats were assessed, and the rats were anaesthetized using isoflurane and sacrificed. Afterwards, their cortexes and hippocampus were rapidly dissected via surgery on ice, and we performed the cerebral biochemical index determination.

### 2.4. Morris Water Maze (MWM)

To assess the recognition memory and spatial learning ability of rats, the Morris water maze test was performed daily for five days as described previously [20]. On the sixth day’s spatial probe experiment, the platform was taken away, and the time of rats spent in the platform quadrant was recorded to measure the maintenance of spatial memory, avoiding the influence of accidental encounters with the platform. In addition, the visible platform test was used to exclude rats with blindness.

### 2.5. Blood Pressure Measurement

As previously described [21], after the behavioral experiments, systolic blood pressure and heart rate in conscious animals were assessed by the tail-cuff method (BP 98A, Softon, Tokyo, Japan) at the end of W1302 or nimodipine treatment. The animal was placed in a constant-temperature incubator at 37 °C for at least 5 min before the measurement; then, the occlusion cuff and volume pressure recording sensor were attached to the tail of the rat. Once the blood volume in the tail was sufficient, the blood pressure and heart rate in each individual rat were determined.

### 2.6. Cell Culture and Treatment

SK-N-SH cells was purchased from the Cell Resource Centre, Institute of Basic Medical Sciences, CAMS and PUMC, and cultured in DMEM (GIBCO) with 10% fetal bovine serum (GIBCO), 100 U/mL penicillin G, and 100 mg/mL streptomycin, at 37 °C, under a 5% CO_2_/95% air environment. When the SKN cells were grown to 70–80% confluence, we harvested the cells and seeded them into 6-well plates at the destiny of 8 × 10^5^ cells/well. To determine the effect of W1302 on the NO/cGMP pathway, the cells were preincubated in the presence of Nω-nitro-L-arginine methyl ester (L-NAME, 10 μM, Sigma, St. Louis, MO, USA; Cat #N5751) and 3-Isobutyl-1-methylxanthine (IBMX, 250 Μm, Sigma, St. Louis, MO, USA; Cat #410957) for 30 min prior to the treatment with W1302 at different concentrations. After 4 h of treatment, the cells were washed twice with ice-cold phosphate-buffered saline (PBS) and lysed with 100 µL of 0.1 M HCL per well. The cell lysates were centrifuged at 12,000× *g* for 10 min at 4 °C, and the supernatants were used for cGMP determination. And the protein concentration was determined by the Bradford method. The cGMP in supernatants was quantified by using an ELISA (enzyme-linked immunosorbent assay) kit (Abcam, Discovery Drive, Cambridge Biomedical Campus, Cambridge, UK) according to the manufacturer’s instructions.

### 2.7. Biochemical Analysis

Tissue treatment was processed according to the manufacturer’s instructions. The contents of malondialdehyde (MDA), NO, and adenosine triphosphate (ATP) in the cortical or hippocampal homogenate of the animals were assayed by a commercial assay kit (Nanjing Jiancheng Bioengineering Research Institute; Nanjing, China). The commercial ELISA kits were purchased from Cozmo-lab Corp. (Shanghai, China) and used for the determination of cyclic guanosine monophosphate (cGMP, Abcam, ab234585), tumor necrosis factor (TNF-α, RayBio^®^, Peachtree Corners, GA, USA, Lot#0104190709), interleukin-1 beta (IL-1β, RayBio^®^, Lot#0118190721), endothelial nitric oxide synthase (eNOS, CUSABIO^®^, Wuhan, China, CSB-E08323r), and inducible nitric oxide synthase (iNOS, CUSABIO^®^, Wuhan, China, CSB-E08325r). A commercial BCA Protein Assay kit (Applygen Technologies Inc., Beijing, China) was used for protein quantification.

### 2.8. Mitochondrial Membrane Potential Measurements

Mitochondrial extraction and membrane potential determination were assessed using a commercial assay kit with JC-1 dye according to the manufacturer’s protocol (Nanjing Jiancheng Bioengineering Research Institute; Nanjing, China). A commercial BCA Protein Assay kit (Applygen Technologies Inc., Beijing, China) was used for protein quantification.

### 2.9. Western Blotting

Tissue lysates were prepared using RIPA lysis buffer (Nanjing Jiancheng Bioengineering Research Institute; Nanjing, China) supplemented with complete EDTA-free protease inhibitor mixtures (Roche, Indianapolis, IN, USA) and PMSF (Nanjing Jiancheng Bioengineering Research Institute; Nanjing, China). Protein samples were separated using 10 or 12% SDS-PAGE and then transferred to PVDF membranes (Millipore, Darmstadt, Germany) at 130 mA for 1–2 h. The incubation was performed as previously described [21]. The blots were visualized using chemiluminescence, scanned using an LAS3000 Fujifilm imaging system (Fujifilm, Tokyo, Japan) and analyzed by densitometric evaluation using Quantity-One software (V4.6.6, Bio-Rad, Hercules, CA, USA). Changes in protein expression were expressed as fold-changes relative to WKY rats. The following primary antibodies were used in the Western blotting experiments: Antibodies against IkBα (10268-1-AP) were obtained from proteintech (Rosemont, IL, USA); NF-κB p65 (#8242), Phospho-NF-κB p65_Ser536_ (#3033), Phospho-IκBα_Ser32_ (#2859), β-Tubulin (#2146) and β-actin (#4970) were obtained from Cell Signaling Technology (Beverly, MA, USA).

### 2.10. Statistical Analysis

Statistical analysis was performed by SPSS version 16.0 software. All the data are expressed as mean ± SEM. The results of behavioral tests were analyzed by two-way repeated measures ANOVA followed by the Bonferroni post hoc test. One-way ANOVA analysis followed by the Tukey post hoc test was performed to analyze statistical difference for multiple group comparisons. A *p*-value less than 0.05 was considered statistically significant.

## 3. Results

### 3.1. W1302 Rescued Spatial Learning and Memory Deficits in SHR-2VO Rats

**Figure 1:**
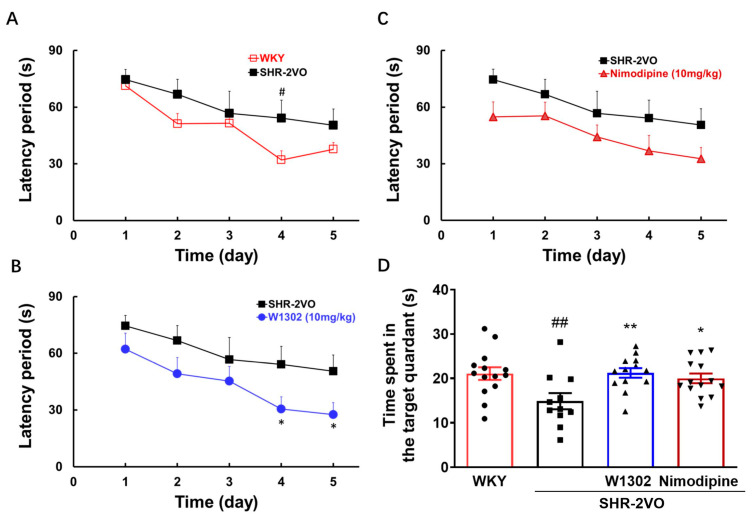
The effect of W1302 on the performance of SHR-2VO rats in the Morris water maze test. SHR-2VO rats: permanent occlusion of bilateral common carotid arteries in spontaneously hypertensive rats; WKY rats: age-matched Wistar Kyoto rats as non-hypertensive sham-operation control. (**A**–**C**) Escape latency for rats to locate the hidden platform in the test during five consecutive days. (**D**) The time of rats spent in the quadrant where the platform was once placed within 90 s. Data were expressed as mean ± S.E.M. (*n* = 11–14). # *p* < 0.05, ## *p* < 0.01 vs. WKY rats and * *p* < 0.05, ** *p* < 0.01, vs. SHR-2VO rats.

The Morris water maze was used to evaluate cognitive function, and the results are shown in Figure 1. SHR-2VO rats exhibited obvious cognitive impairment, which was evidenced by the escape latency to find the hidden platform being distinctly longer (on day 4, *p* < 0.05) than that of WKY rats in a place navigation experiment (Figure 1A). In contrast, W1302 (10 mg/kg) intervention obviously reduced the time to find the hidden platform (on days 4 and 5, *p* < 0.05) (Figure 1B), indicating that W1302 was effective in rescuing a spatial learning deficit in SHR-2VO rats. Meanwhile, nimodipine (10 mg/kg) treatment also showed a decreasing tendency in the time to find the platform (Figure 1C). Furthermore, the spatial probe experiment was performed on the sixth day (Figure 1D). SHR-2VO rats spent a markedly shorter time in the target quadrant than WKY rats (*p* < 0.01). W1302 or nimodipine-treated rats could stay longer in the target quadrant than SHR-2VO rats (*p* < 0.05). These results suggested that W1302 had potential therapeutic effects on VaD induced by SHR-2VO rats.

### 3.2. W1302 Slightly Lower Systolic Blood Pressure (SBP) of SHR-2VO Rats

As shown in Table 1, SHR-2VO rats showed a significant increase in systolic blood pressure by 58% (*p* < 0.01) and heart rate by 15% (*p* < 0.01) compared to WKY rats, respectively. During W1302 or nimodipine treatment, there was no significant alteration in the heart rate relative to that of SHR-2VO rats. Moreover, there was a slight reduction in SBP (6%, *p* > 0.05) in SHR-2VO rats after W1302 treatment compared with that of the SHR-2VO rats. However, nimodipine intervention leads to an apparent decrease in the SBP (20%, *p* < 0.01) of SHR-2VO rats.

### 3.3. W1302 Reversed Mitochondrial Dysfunction and Oxidative Stress in Brain of SHR-2VO Rats

**Figure 2:**
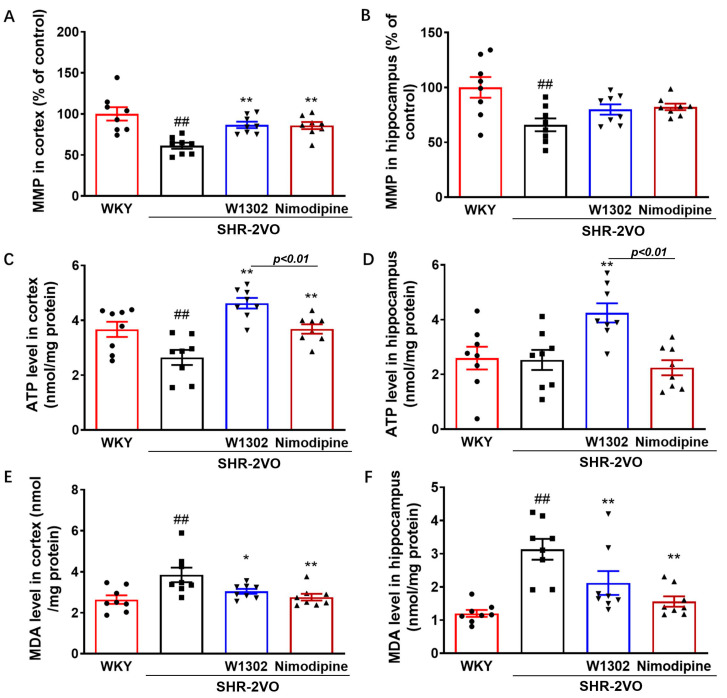
W1302 restored mitochondrial function and attenuated oxidative stress in SHR-2VO rats. SHR-2VO rats: a permanent occlusion of bilateral common carotid arteries in spontaneously hypertensive rats; WKY rats: age-matched Wistar Kyoto rats as non-hypertensive sham-operated control. (**A**,**B**) Mitochondrial membrane potential (MMP, fold increase vs. that of WKY rats) in cortex and hippocampus, (**C**,**D**) ATP levels in cortex and hippocampus, (**E**,**F**) MDA concentrations in cortex and hippocampus. Data were expressed as mean ± S.E.M. (*n* = 8). ## *p* < 0.01 vs. WKY rats and * *p* < 0.05, ** *p* < 0.01, vs. SHR-2VO rats.

Chronic hypertension and cerebral hypoperfusion could alter cerebral blood flow and microvascular pressure, thus damaging mitochondria and increasing oxidative stress [2,22]. As illustrated in Figure 2, SHR-2VO rats showed markedly decreased mitochondrial membrane potential (MMP) (39% in cortex, *p* < 0.01; 34% in hippocampus, *p* < 0.01), ATP production (28% in cortex, *p* < 0.05), and increased MDA levels (46% in cortex, *p* < 0.05; 160% in hippocampus, *p* < 0.01), compared to WKY rats. W1302 or nimodipine treatment attenuated mitochondrial dysfunction and oxidative stress in SHR-2VO rats, as indicated by enhanced MMP (41% for W1302 in cortex, *p* < 0.01; 40% for nimodipine in cortex, *p* < 0.01) (Figure 2A), ATP production (75% or 68% for W1302 in cortex or hippocampus, *p* < 0.01; 39% for nimodipine in cortex, *p* < 0.01) (Figure 2C,D), and decreased MDA levels (21% or 32% for W1302 in cortex or hippocampus, *p* < 0.05; 28% or 50% for nimodipine in cortex or hippocampus, *p* < 0.01) (Figure 2E,F). Compared with nimodipine treatment, W1302 intervention significantly increased ATP production (*p* < 0.01) and improved the energy supply in the brain of SHR-2VO rats (Figure 2C,D).

### 3.4. W1302 Increased NO Release and Inhibited iNOS Activity in Brain of SHR-2VO Rats

**Figure 3:**
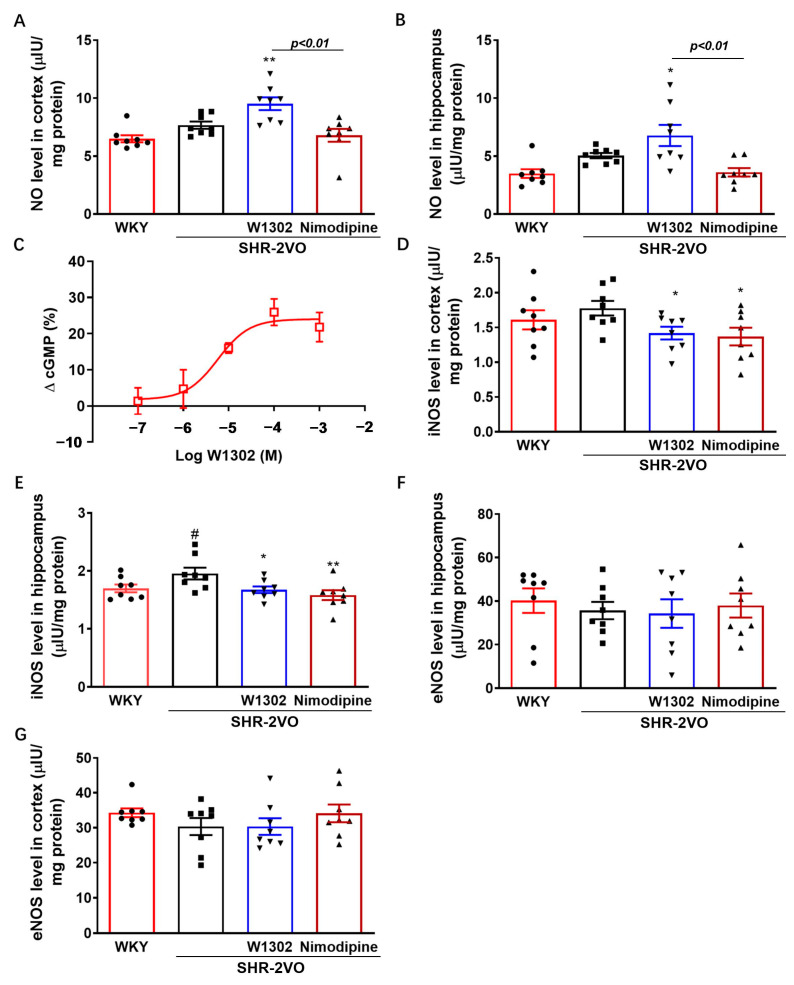
The effects of W1302 on NO production and activities of eNOS or iNOS in SHR-2VO rats. SHR-2VO rats: permanent occlusion of bilateral common carotid arteries in spontaneously hypertensive rats; WKY rats: age-matched Wistar Kyoto rats as non-hypertensive sham-operated control. (**A**,**B**) The nitric oxide assay kit for detecting the production of NO (*n* = 8). (**C**) EC_50_ value of cGMP production in SKN cells after 4 h of incubation with W1302 (*n* = 4). (**D**–**G**) eNOS and iNOS were analyzed by Elisa kits (*n* = 8). Data were expressed as mean ± S.E.M. # *p* < 0.05 vs. WKY rats and * *p* < 0.05, ** *p* < 0.01, vs. SHR-2VO rats.

W1302 is a nitric oxide donor, and its effects on NO production were observed by measuring nitrite formation in the brain of rats. Levels of nitrite in cortex and hippocampus exhibited an increasing tendency in SHR-2VO rats relative to WKY rats. Figure 3A,B showed that W1302 increased nitrite production in the cortex by 24% (*p* < 0.01) and in the hippocampus by 34% (*p* < 0.05), respectively, compared with that of SHR-2VO rats. However, the nimodipine treatment group showed no difference in the nitrite production relative to SHR-2VO rats. Also, compared with nimodipine treatment, W1302 intervention significantly increased NO production (*p* < 0.01) in the brains of SHR-2VO rats. To investigate whether the increase in nitrite production was translated into the activation of the endogenous NO-cGMP pathway, cGMP production was determined in SKN cells after 4 h of incubation with W1302 at different concentrations. As seen in Figure 3C, the effect of W1302 on cGMP production in SKN cells was observed with the EC_50_ of 5.7 ± 2.1 μM.

In addition, NO is synthesized by nitric oxide synthase (NOS), of which there are three known isoforms: nNOS, eNOS, and iNOS. The effects of W1302 on the NOS activity of brain tissue are shown in Figure 3D–G. Levels of iNOS in the hippocampus significantly increased by 15% (*p* < 0.05), whereas the cortex exhibited only an increasing tendency in SHR-2VO rats relative to WKY rats. The W1302 treatment group showed dramatically decreased iNOS activity by 20% (*p* < 0.05) and 14% (*p* < 0.05) in the cortex and hippocampus, respectively. The nimodipine treatment group also showed significantly reduced iNOS activity by 23% (*p* < 0.01) and 19% (*p* < 0.05) in the cortex and hippocampus, respectively. But there were no significant differences in the eNOS activity among all groups.

### 3.5. W1302 Inhibited Inflammation through NF-κB Signal Pathway in Brain of SHR-2VO Rats

**Figure 4:**
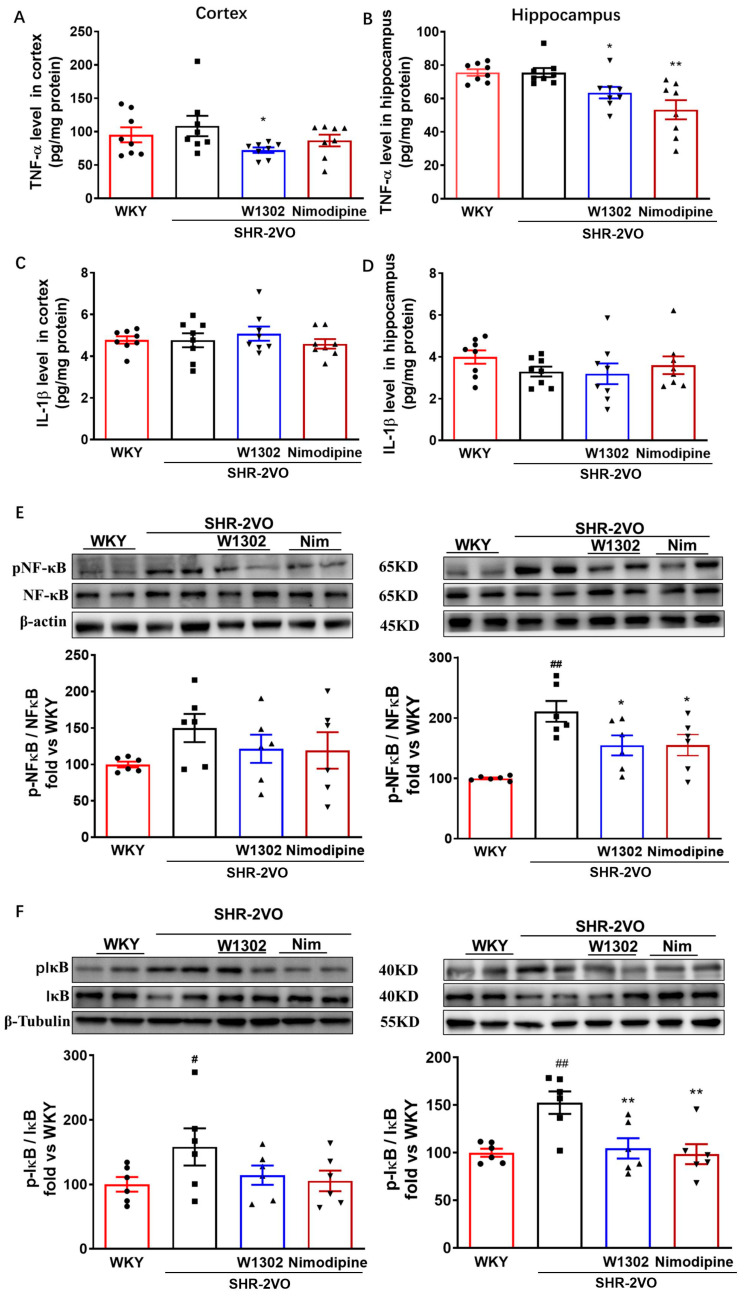
Effects of W1302 on the expression of inflammatory factors and the NF-κB pathway in SHR-2VO rats. SHR-2VO rats: permanent occlusion of bilateral common carotid arteries in spontaneously hypertensive rats; WKY rats: age-matched Wistar Kyoto rats as non-hypertensive sham-operated control. Quantitative analysis of TNF-α (**A**,**B**) and IL-1β (**C**,**D**) levels by Elisa kits (*n* = 8). (**E**) Representative bands of Western blot and quantitative analysis of NF-κB phosphorylation level and NF-κB expression (*n* = 6). (**F**) Representative bands of Western blot and quantitative analysis of IκBα phosphorylation level and IκBα expression (*n* = 6). Data were expressed as mean ± S.E.M. # *p* < 0.05, ## *p* < 0.01 vs. WKY rats and * *p* < 0.05, ** *p* < 0.01, vs. SHR-2VO rats.

On the other hand, increasing evidence suggests that NO plays a key role in protecting blood vessels from inflammation [23,24,25]. In our study, the pro-inflammatory cytokines levels of TNF-α and IL-1β showed no obvious differences in the SHR-2VO rats (Figure 4A–D), whereas the expressions of pNF-κB and pIκBα were significantly increased in the brains of SHR-2VO rats compared with those of WKY rats (Figure 4E,F). The administration of W1302 rescued hypoperfused-induced injury in SHR-2VO rats, as indicated by lower pIκBα/IκBα and pNF-κB/NF-κB expression by 31% (*p* < 0.01) and 27% (*p* < 0.05) in the hippocampus, respectively (Figure 4E,F), as well as decreased TNF-α levels by 20% (*p* < 0.05) in the cortex and 19% (*p* < 0.05) in the hippocampus, respectively (Figure 4A,B). The nimodipine treatment group showed a similar effect to W1302 intervention. The results suggested that W1302 might regulate NF-κB activation to inhibit inflammation such as TNF-α.

## 4. Discussion

Hypertension plays a critical role in the pathogenesis of VaD [2]. It affects about 30% of older adults (aged > 60 years) and markedly increases the risk of vascular cognitive decline [1,2,3,4,5]. VaD is emerging as a serious public health problem all around the world due to its high mortality and disability rates [2,3]. Hypertension reduces the bioavailability of vascular NO, accelerates brain atrophy, promotes inflammation and the dysfunction of cerebral microcirculation as well as microvascular endothelial, impairs cerebral blood supply, and compromises the blood–brain barrier. These alterations lead to the onset of neurodegenerative diseases, such as VaD [5,6,7,9,10].

SHRs are usually used to elucidate the deleterious effects of uncontrolled high blood pressure on the brains [26]. In the present study, we used the permanent occlusion of a bilateral common carotid arteries model related to spontaneous hypertension to examine the complications in VaD, which showed the progressive and long-term learning and memory deficits. And nimodipine was served as a positive drug, which had a neuroprotective effect via regulating the NF-κB signaling pathway in rats after focal cerebral ischemia injury previously [19]. After 4 weeks of treatment, W1302 significantly ameliorated the learning and memory impairment in SHR-2VO rats with a slight decrease in systolic blood pressure by 6%. Our previous studies also observed that W1302 significantly reduced the SBP in SHR rats by 10.5% (173.7 ± 4.0 mmHg in W1302-treated SHR rats vs. 194.1 ± 4.2 mmHg in SHR rats). In addition, nimodipine significantly reduced SBP by 20% and tended to improve the learning and memory impairment in SHR-2VO rats, which was consistent with our previous studies of 2VO rats [27]. These findings suggest that the neuroprotective effect of W1302 in SHR-2VO rats is better than that of nimodipine, and its mechanism of action is different from that of nimodipine and may not be mediated primarily by blood pressure reduction.

Chronic hypertension and cerebral hypoperfusion can reduce cerebral blood flow, impair microvascular endothelium, thereby damaging mitochondria, and increase oxidative stress [2,22]. It has been documented that oxidative stress was involved in various neurodegenerative disorders, including VaD [6]. Mitochondria are the predominant source of energy; however, under pathological conditions such as hypertension, they lead to mitochondrial dysfunction that causes severe energy deficiency and endogenous reactive oxygen species (ROS) overproduction, which are capable of mediating the neuronal degeneration and death involved in the pathogenesis of VaD [2,6]. In this study, we observed mitochondrial dysfunction and oxidative stress, which was indicated by impaired MMP, decreased ATP production, and increased MDA levels in the cortex and hippocampus of the SHR-2VO rats. Moreover, NOD has also been proven to inhibit oxidative stress. The treatment of W1302 showed significant effects on reversing these abnormal alterations in the cortex and hippocampus. In contrast, nimodipine also restored these aberrant changes, suggesting that W1302 has a similar effect on mitochondrial protection in restoring energy supply and inhibiting oxidative stress, but its ATP production is superior to that of nimodipine.

It is well known that NO is associated with cognitive function, and it plays a dual role, that is, neuroprotection and neurotoxicity [25]. NO is synthesized by nitric oxide synthase (NOS), of which there are three known isoforms: nNOS, eNOS, and iNOS. The baseline concentration of NO in the brain is mainly due to nNOS activity and secondarily to eNOS. The iNOS level is low under physiological conditions [28]. It has been demonstrated that eNOS-derived NO scavenges ROS and inhibits the expression of cellular adhesion molecules, platelet aggregation, and leukocyte adhesion [29]. eNOS knockout mice were more susceptible to ischemia–reperfusion injury with larger infarcts and a greater reduction in cerebral blood flow [30]. Conversely, iNOS-produced NO contributes to brain injury. iNOS expression is increased in the microglia and astrocytes cells following events such as inflammatory, trauma, and immune responses. iNOS knockout in AD transgenic mice or iNOS inhibitors could block NO production and show a protective effect against Aβ-induced neurotoxicity [31]. In our study, SHR-2VO rats showed a slight increase in NO levels and iNOS activity in the hippocampus and cortex. This was compatible with previous studies in which during the initial stage of ischemia, NO concentration decreased because of oxygen deficiency and subsequently increased due to iNOS expression [32]. Chronic treatment with W1302 showed a significantly increase in NO level and a distinct decline in the iNOS activity of the hippocampus and cortex of the SHR-2VO rats. W1302 is a NO donor, and the increase in NO or NO-related species in the brain may be independent of its endogenous sources. Nimodipine treatment also restored the iNOS activity in SHR-2VO rats, but the NO level was not significantly improved, which was consistent with previous studies [19].

There is evidence showing that iNOS is secondary to inflammation such as TNF-α and IL-1β as well as oxidative stress [25]. During neuro-inflammation, NF-κB is localized in the nucleus by the phosphorylation of IκBα to promote the transcription of pro-inflammatory cytokines (TNF-α IL-6, IL-1β, iNOS, COX-2) and activate an inflammatory response [2]. Our results showed that the levels of pNF-κB, pIκB, and TNF-α were increased significantly in the cortex and hippocampus of SHR-2VO rats. W1302 or nimodipine treatment attenuated the pNF-κB and pIκBα expression and in response reduced the levels of TNF-α markedly, signifying the potential of W1302 to inhibit the activation of the NF-κB signaling pathway. Nimodipine has the same effect, which is consistent with previous reports [19].

## 5. Conclusions

**Figure 5:**
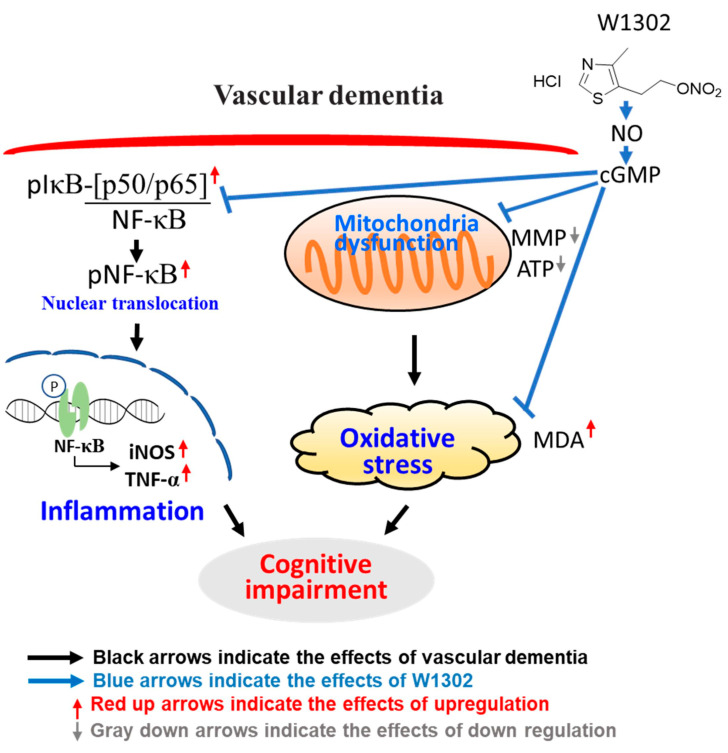
A schematic illustration of W1302 alleviating learning impairments induced by hypoperfusion with hypertension through the NO-regulated NF-κB signaling pathway and mitochondrial function preservation. In this schematic diagram, SHR-2VO rats, a VaD model associated with spontaneous hypertension, significantly increase pIκB, pNF-κB, iNOS and TNF-α levels, which further damage mitochondrial function and increases oxidative stress. W1302 activated NO/cGMP and further inhibited the NF-κB signaling pathway, decreasing inflammatory factors such as TNF-α and iNOS levels, and protecting cells from mitochondria damage.

To summarize, a schematic model summarizing the mechanisms elucidated by our study is provided in Figure 5. W1302 may suppress hypoperfusion with hypertension-induced learning impairments through the NO-regulated NF-κB signaling pathway, decrease the expression of TNF-α, and preserve mitochondrial function. These results suggest that W1302 may be a promising agent for the treatment of VaD. Further investigations are still needed to validate the neuroprotective effects of W1302 against other VaD models before the clinical trials, whereas the stronger NO bioavailability and ATP production by W1302 may explain the difference in neuroprotection from that of nimodipine.

The study has some potential limitations. Due to the unexpectedly high mortality (~45%) in surgical animals, we performed experiments using single-dose SHR-2VO rats to explore the neuroprotective effects of W1302 administration; gender (especially female animals) and cerebral blood flow were also not considered as variables in the present study.

## Figures and Tables

**Table 1:** Systolic blood pressure (SBP) and heart rate of WKY and SHR-2VO rats after W1302 or nimodipine treatment.

Groups	Dose (mg/kg)	N	Heart Rate (bpm)	SBP (mmHg)
WKY	−	16	296 ± 9	129 ± 3
SHR-2VO	−	11	341 ± 14 ##	204 ± 5 ##
SHR-2VO + nimodipine	10	15	375 ± 14	164 ± 3 **
SHR-2VO + W1302	10	13	351 ± 11	192 ± 4

SHR-2VO rats: permanent occlusion of bilateral common carotid arteries in spontaneously hypertensive rats; WKY rats: age-matched Wistar Kyoto rats as non-hypertensive sham-operated control. Data were expressed as mean ± S.E.M. ## *p* <0.01, vs. WKY group, ** *p* <0.01, vs. SHR-2VO group.

## Data Availability

The data are contained in the article.
